# Surgical treatment for mediastinal parathyroid adenoma causing primary hyperparathyroidism

**DOI:** 10.1186/s13019-016-0461-8

**Published:** 2016-04-07

**Authors:** Masahiro Kitada, Shunsuke Yasuda, Takahashi Nana, Kei Ishibashi, Satoshi Hayashi, Satoru Okazaki

**Affiliations:** Department of Breast Disease Center, Asahikawa Medical University, Midorigaoka-Higashi 2-1-1-1, Asahikawa, Hokkaido 078-8510 Japan

## Abstract

**Background:**

Primary hyperparathyroidism is a rare disease characterized by excessive secretion of parathyroid hormone from parathyroid adenoma, hyperplasia, or malignancy. The clinical symptoms of the condition are those of hypercalcemia. Although the lesions are commonly located in the neck region, in about 1–2 % of cases, the lesions are ectopically located within the mediastinum, where surgical excision using the cervical approach is difficult. The principal treatment of the condition is surgical excision of the lesion. However, some patients require additional surgery because of recurrence due to intraoperative dissemination. Therefore, safe and accurate excision is essential for the treatment. We reviewed the surgical treatment used at our institution for mediastinal parathyroid adenoma that caused primary hyperparathyroidism.

**Method:**

The subjects were four patients with primary hyperparathyroidism due to mediastinal parathyroid adenoma who underwent surgery at our institution within a period of 10 years, between January 2005 and December 2014. All of the patients were female, with a mean age of 64.5 years (range, 55–74 years). The examined variables included background factors, clinical condition, surgical method, and clinical outcome.

**Result:**

In all of the patients, primary hyperparathyroidism was detected with symptoms of hypercalcemia. Laboratory tests revealed a mean serum calcium level of 11.85 mg/dL (range, 11.2–13.2 mg/dL) and a mean parathyroid hormone (intact PTH) level of 304.8 pg/mL (range, 126–586 pg/mL), indicating elevated levels for all patients. Chest computed tomography (CT) revealed tumors with a mean diameter of 2.8 cm (range, 10–45 mm) in the anterior mediastinum in all of the patients. On 99mTC-methoxy isobutyl isonitrile (MIBI) scintigraphy, abnormal accumulation was observed in all of the patients. Regarding the surgical methods, median sternotomy was used for three cases and upper partial sternotomy was used for one case. The surgery was safely and accurately performed, without postoperative complications. After surgery, the serum calcium levels immediately returned to normal and the symptoms improved.

**Conclusion:**

We performed excision safely and accurately in all of the patients. In tumor identification, 99mTC-MIBI scintigraphy was useful. Accurate tumor identification and selection of the optimal surgical method are important for prevention of recurrence due to intraoperative dissemination.

## Background

Primary hyperparathyroidism is a disease with various symptoms related to hypercalcemia due to excessive secretion of parathyroid hormone from a tumor or hyperplasia, such as adenoma and carcinoma, which arises in the parathyroid. The principal treatment of the condition is surgical excision of the pathological tissue. Although most parathyroid adenomas are located in the neck region, 11–25 % of them are located within the mediastinum and 1–2 % are located in the mediastinum, where surgical excision using the cervical approach is difficult [1–3]. We experienced surgical treatment of a mediastinal parathyroid adenoma that caused primary hyperparathyroidism in four patients.

## Method

The subjects were four patients with primary hyperparathyroidism due to mediastinal parathyroid adenoma who underwent surgery at our institution within a period of 10 years, between January 2005 and December 2014. All of the patients were female, with a mean age of 64.5 years (range, 55–74 years). The examined variables included background factors, symptoms at the time of diagnosis, clinical condition, surgical method, and clinical outcome.

## Result

**Fig. 1:**
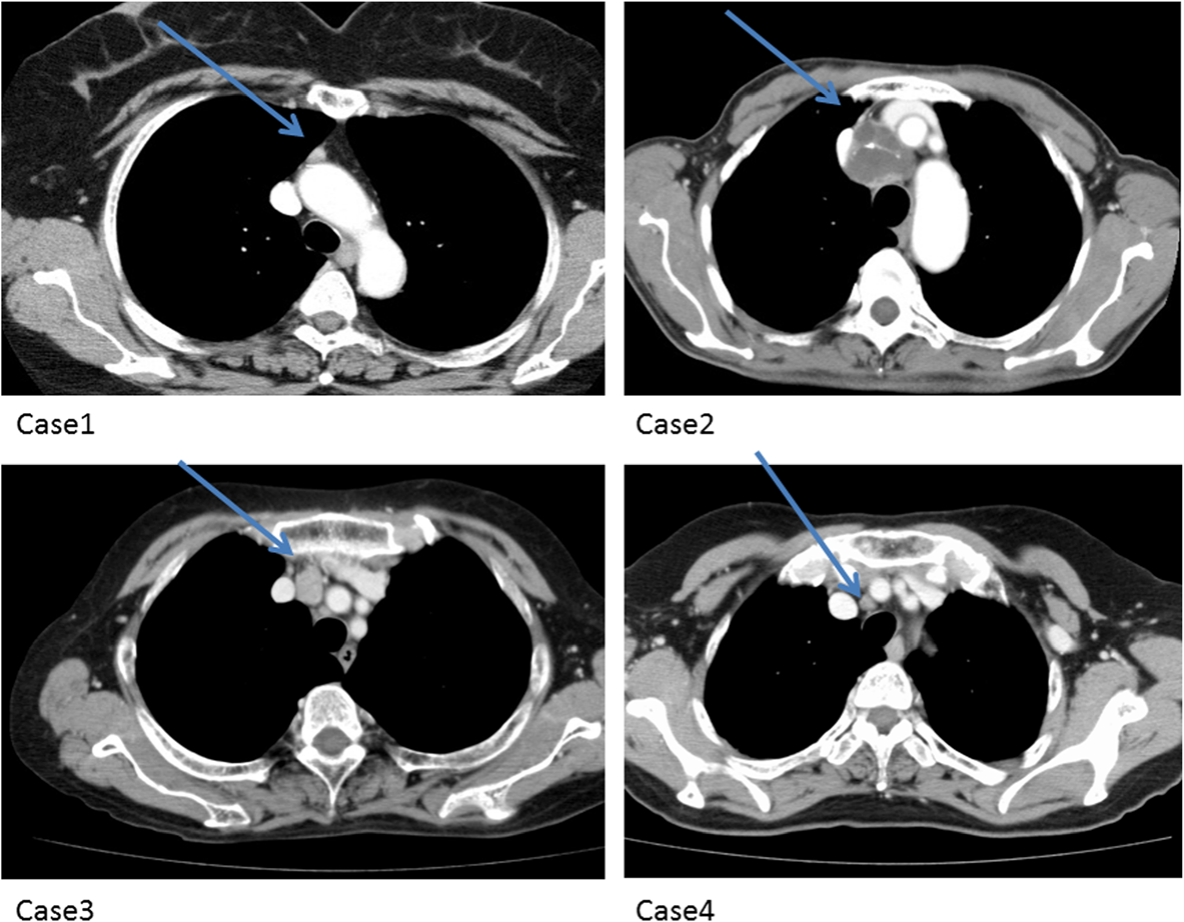
Computerized tomography. Mediastinum parathyroid adenoma or hyperplasia legion (arrow)

**Fig. 2:**
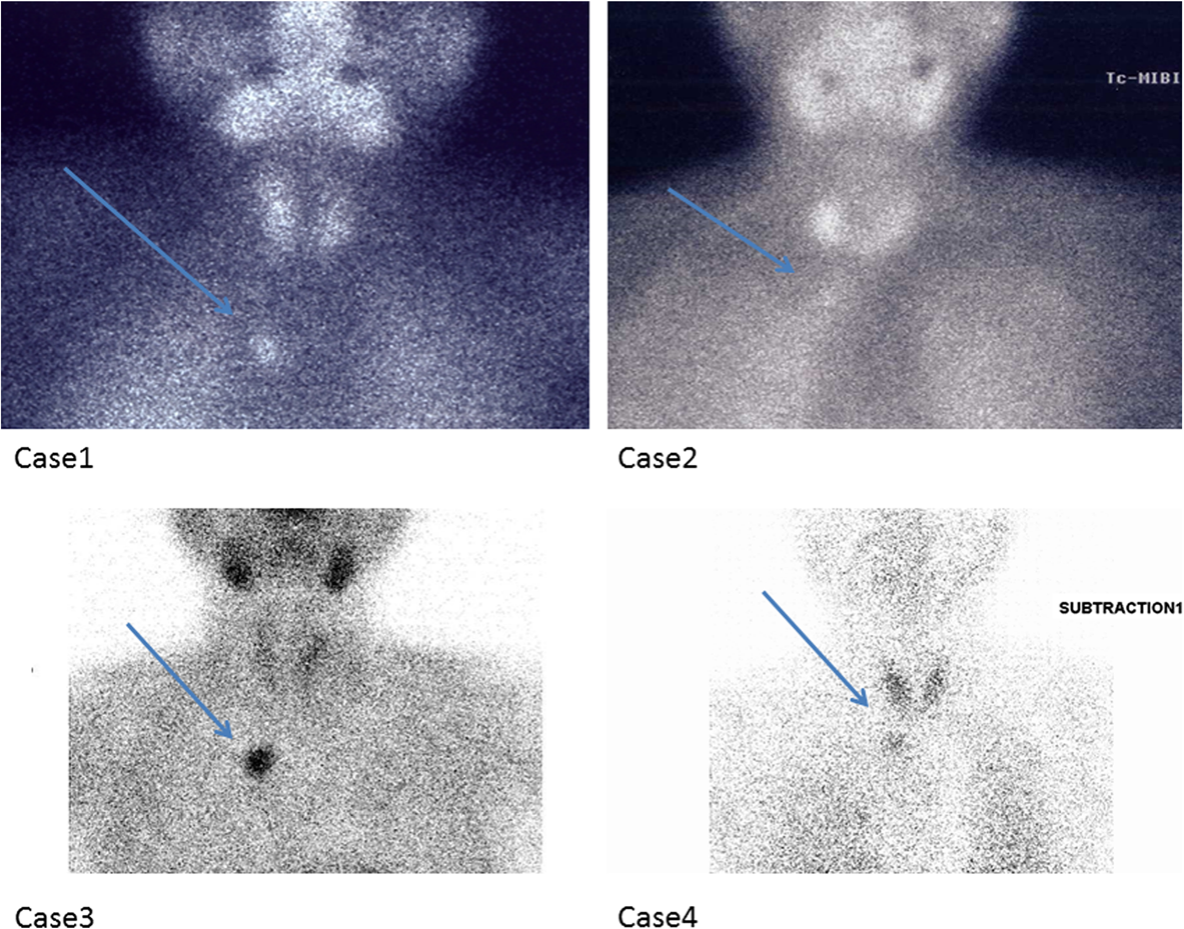
99mTc MIBI scan. Mediastinum parathyroid adenoma or hyperplasia lesion (arrow). Image of case3 and 4 are subtraction image

The clinical symptoms of and surgical methods used for all the patients are shown in Table 1. In all of the patients, primary hyperparathyroidism was detected with the following symptoms of hypercalcemia: two patients presented with weakness, including muscle strength reduction; one presented with urinary calculus; and the remaining patient presented with digestive symptoms such as nausea and abdominal pain. Laboratory tests showed a mean serum calcium level of 11.85 mg/dL (range, 11.2–13.2 mg/dL) and a mean intact PTH level of 304.8 pg/mL (range, 126–586 pg/mL), indicating elevated levels in all of the patients. Chest CT revealed tumors with a mean diameter of 2.8 cm (range, 10–45 mm) in the anterior mediastinum in all of the patients. One patient had a cystic tumor (case 3; Fig. 1). On 99mTC-MIBI scintigraphy, abnormal accumulation was observed in all of the patients (Fig. 2). Regarding the surgical methods, median sternotomy was used for cases 1, 2, and 3, and upper partial sternotomy was used for case 4. The surgery was safely and accurately performed. After surgery, the serum calcium levels immediately returned to normal, and the symptoms improved without recurrence. According to histological examination results, three patients were diagnosed with parathyroid adenoma and the remaining patient was diagnosed with parathyroid hyperplasia. None of the patients had any malignancy. Among the three patients with adenoma, one had a cystic mass.

**Table 1:** Characteristics of patients with hyperthyroidism in whom mediastinal lesions and surgical approach, pathology, clinical outcome

Case	1	2	3	4
Age	57	72	74	55
Gender	women	women	women	women
Chief complaint	nephrolithiasis	listlessness	listlessness	abdominal pain feel nausea
Serum calcium level (mg/dl)	11.1	11.2	13.2	11.9
intact-PTH (pg/dl)	126	353	586	154
Location	anterior mediastinum	anterior mediastinum	anterior mediastinum	anterior mediastinum
Surgical approach	Total sternotomy	Total sternotomy	Total sternotomy	Partial sternotomy
Pathology	adenoma	adenoma	adenoma	hyperplasia
Post operative hyperthyroidism	cured	cured	cured	cured

## Discussion

Most cases of primary hyperparathyroidism are caused by excessive secretion of intact PTH from parathyroid adenoma or parathyroid hyperplasia. About 90 % of patients are women. The mean age of the patients is reportedly around 60 years. The disease is usually detected with symptoms of hypercalcemia, including urinary calculus, bone lesion (osteoporosis), polyposia and polyuria, general malaise, vomiting, and constipation. Recently, in a number of patients, the disease has been detected incidentally during routine medical checkup, according to laboratory test results that show hypercalcemia, even without typical clinical symptoms. The condition can occur as a part of multiple endocrine neoplasia type 1 (MEN1), which consists of primary hyperparathyroidism, gastroenteropancreatic neuroendocrine tumor, and pituitary adenoma [4, 5]. Most parathyroid adenomas are located in the neck region and mediastinum. In particular, 11–25 % of parathyroid adenomas are located within the mediastinum, of which about 2 % are difficult to excise by using the cervical approach [1–3]. In addition, mediastinal parathyroid adenomas are frequently small; therefore, accurate localization is essential [6]. In addition, 99TC-MIBI scintigraphy is useful for localization of the tumor and therefore essential for preoperative diagnosis [1, 7]. Previous studies also reported the usefulness of longitudinal vein harvesting [8, 9] and monitoring of serum PTH (intraoperative PTH) levels [10].

While a study reported a successful treatment of the condition with percutaneous ethanol injection, [11] most cases require tumor excision. Conventionally, median sternotomy has been used for surgical excision of mediastinal parathyroid adenomas that are difficult to excise using the cervical approach. This method has advantages of accuracy in tumor identification and good operative view. However, given that most of the tumors are benign, less-invasive approaches have gained popularity. In addition to cases of upper partial sternotomy, cases of thoracoscopic excision [12, 13] and excision through parasternal intercostal incision have been reported [14]. Therefore, selection of the optimal surgical approach for each patient is important for accurate tumor identification and excision.

## Conclusion

We reviewed the surgical treatment used in our institution for four cases of ectopic mediastinal parathyroid adenoma that caused primary hyperparathyroidism. Accurate tumor identification and selection of the optimal surgical method are important for prevention of recurrence due to intraoperative dissemination.

## Consent

Informed consent was obtained from each patient for publication. A copy of the written consent is available for review by the Editor-in Chief of this journal
